# The effects of mechanical stretch on the biological characteristics of human adipose‐derived stem cells

**DOI:** 10.1111/jcmm.14314

**Published:** 2019-04-24

**Authors:** Bin Fang, Yanjun Liu, Danning Zheng, Shengzhou Shan, Chuandong Wang, Ya Gao, Jing Wang, Yun Xie, Yifan Zhang, Qingfeng Li

**Affiliations:** ^1^ Department of Plastic and Reconstructive Surgery Shanghai Ninth People's Hospital, Shanghai Jiao Tong University School of Medicine Shanghai China; ^2^ Department of Orthopedic Surgery Xin Hua Hospital Affiliated to Shanghai Jiao Tong University School of Medicine Shanghai China; ^3^ Department of Otorhinolaryngology and Head & Neck Surgery Shanghai Children's Hospital, Shanghai Jiao Tong University Shanghai China

**Keywords:** adipose‐derived stem cells, biological characteristics, cell therapy, mechanical stretch, mesenchymal stem cell, tissue regeneration

## Abstract

Adipose‐derived stem cells (ADSCs) are a subset of mesenchymal stem cells (MSCs), which have promised a vast therapeutic potential in tissue regeneration. Recent studies have demonstrated that combining stem cells with mechanical stretch may strengthen the efficacy of regenerative therapies. However, the exact influences of mechanical stretch on MSCs still remain inconclusive. In this study, human ADSCs (hADSCs) were applied cyclic stretch stimulation under an in vitro stretching model for designated duration. We found that mechanical stretch significantly promoted the proliferation, adhesion and migration of hADSCs, suppressing cellular apoptosis and increasing the production of pro‐healing cytokines. For differentiation of hADSCs, mechanical stretch inhibited adipogenesis, but enhanced osteogenesis. Long‐term stretch could promote ageing of hADSCs, but did not alter the cell size and typical immunophenotypic characteristics. Furthermore, we revealed that PI3K/AKT and MAPK pathways might participate in the effects of mechanical stretch on the biological characteristics of hADSCs. Taken together, mechanical stretch is an effective strategy for enhancing stem cell behaviour and regulating stem cell fate. The synergy between hADSCs and mechanical stretch would most likely facilitate tissue regeneration and promote the development of stem cell therapy.

## 1 INTRODUCTION

Recent advances in regenerative medicine, particularly the discovery of mesenchymal stem cells (MSCs), which exhibit pro‐healing and immunomodulatory effects,^1^ have provided the opportunity of using autologous stem cell transplantation to promote tissue repair and regeneration in clinical practice.^2^ Adipose‐derived stem cells (ADSCs) are multipotent stem cells obtained from adipose tissue, which are a subset of MSCs and possess the capacity for self‐renewal and multi‐lineage differentiation.^3^ Compared with other kind of MSCs, ADSCs are ubiquitous and can be easily harvested with minimal donor site morbidity,^4^ which have led to a burgeoning field of research. In addition, ADSCs have high yields, great proliferative rates in culture and weak immunogenicity.^5^, ^6^ In consequence, ADSCs could be considered as a kind of ideal seed cell for translational medicine research and regenerative medicine therapies.

Mechanical stretch is one of physical cues from an organismal level to a subcellular level, which is associated with many physiological and pathological processes such as beating heart or bone remodelling processes.^7^, ^8^ It has been indicated that in vitro mechanical stretch can modulate the balance of self‐renewal and differentiation in various cell types. For example, cyclic stretch induces increased proliferation of human skin keratinocytes and dermal fibroblasts.^9^, ^10^ It also induces the cardiogenesis and vasculogenesis of embryonic stem cells (ESCs).^11^, ^12^ It is noteworthy that mechanical stretch has been widely used as an important newfangled pretreatment method in tissue engineering. For instance, cyclic stretch preconditioning improves engineered muscle contraction.^13^ Proper mechanical stimulation may promote the cell‐based ligament regeneration and success rate of transplantation.^14^ Therefore, mechanical stretch may promote tissue regeneration by influencing cell biological characteristics.

In plastic and reconstructive surgery, ADSCs have been used for a variety of different applications such as autologous fat grafting or treatment of chronic wounds.^15^, ^16^ On the other hand, tissue regeneration through continuous mechanical stretch has received widely recognized during the last decades for repair of soft tissue defects.^17^ Recent studies have found that combining stem cells with mechanical stretch may strengthen the effect of tissue regeneration. It is reported that the transplantation of ADSCs could possibly promote mechanical stretch induced skin regeneration and mitigate traditional limitations of skin expansion.^18^ ADSCs also play a key role in external volume expansion induced by negative pressure.^19^ When primed with stiff substrates, ADSCs stimulated by mechanical stimulus exhibited increased therapeutic efficacy in pathological wound healing.^20^ In conclusion, mechanical stretch could enhance the therapeutic efficacy of ADSCs, and ADSCs has synergistic effect with mechanical stretch in promoting tissue regeneration. However, the exact influences of mechanical stretch on ADSCs still remain inconclusive.

Given this, our study aimed to investigate the effects of mechanical stretch on the biological characteristics of human ADSCs (hADSCs). In this study, hADSCs were applied cyclic stretch stimulation under an in vitro stretching model and the cellular proliferation, apoptosis, adhesion, migration, cytokine production, as well as differentiation potentials of hADSCs were examined. We further investigated the phenotypic characteristics of hADSCs cultured under cyclic stretch stimulation during long‐term cultivation and mechanical stretch related cell signalling events.

## 2 MATERIALS AND METHODS

### 2.1 Patients and samples

Subcutaneous adipose tissues were obtained from five healthy donors who underwent abdominal liposuction with informed consents of Shanghai Ninth People's Hospital. This study was approved by the Institutional Ethics Committee of Shanghai Ninth People's Hospital.

### 2.2 Isolation and culture of hADSCs

The adipose tissue was minced and digested with 0.2% collagenase type I (Sigma‐Aldrich, USA) at 37°C for 1 hour. After filtration, centrifugation and resuspension, the pelleted hADSCs were treated in Dulbecco's Modified Eagle Medium (DMEM) (Gibco, Life Technique, NY) supplemented with 10% foetal bovine serum (FBS) (Hyclone, Thermo Fisher, MA) and 1% penicillin/streptomycin (Gibco, Life Technique, NY). After 48 hours, the non‐adherent cells were removed and the medium was then changed every 3 days. The adherent spindle‐shaped cells were further propagated for three passages.

### 2.3 Immunophenotypic analysis of hADSCs

For flow cytometric analysis, P3 hADSCs were incubated with monoclonal PE‐conjugated antibodies for CD34, CD45 and HLA‐DR or with FITC‐conjugated antibodies for CD73, CD90 and CD105 at room temperature for 30 min (BD Pharmingen, San Diego, CA). Isotype control IgG was used to stain the cells as control. The cells were subsequently washed with phosphate‐buffered saline (PBS), fixed with 4% formaldehyde and analysed on a FACScan flow cytometer (Becton‐Dickinson, San Jose, CA).

### 2.4 Multipotential induction

Multilineage differentiation capacity of hADSCs was detected as requested by the International Society for Cellular Therapy. Briefly, cells were cultured in adipogenic induction medium (Cyagen Biosciences, USA) for designated time and were stained by Oil Red‐O staining. For osteogenic differentiation, cells were cultured in osteogenic inducing medium (Cyagen Biosciences, USA) for designated time and were stained by alizarin red S. Chondrogenic differentiation was performed using the micromass culture technique maintained in chondrogenic medium (Cyagen Biosciences, USA) for up to 5 weeks, and were stained by Alcian blue.

### 2.5 Application of mechanical stretch

**Figure 1:**
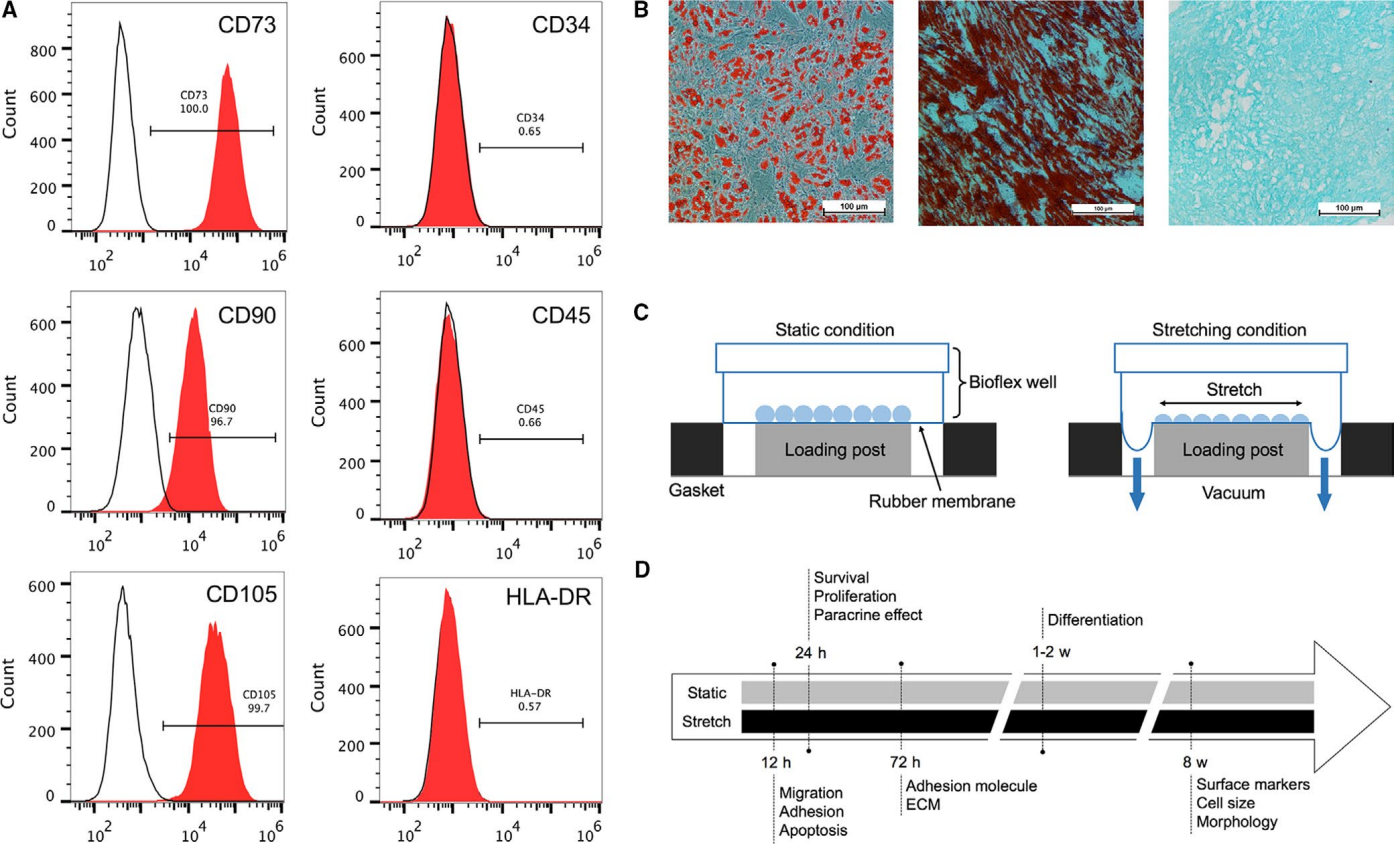
Characterization of human adipose‐derived stem cells (hADSCs) and experiment design. (A) Immunophenotypic analysis was performed by flow cytometry. (black line: isotype control, red line: sample). (B) hADSCs are able to differentiate to adipogenic, osteogenic and chondrogenic lineages in vitro, as demonstrated by positive oil red O staining, alizarin red S staining and Alcian blue staining. Scale bar: 100 μm. (C) Diagram of side view of Flexcell Tension system. (D) Experimental designs and time schedule of assessments

Mechanical stretch was applied to hADSCs by FX‐5000T™ Flexcell Tension Plus (Flexcell International Corporation, Hillsborough, NC) (Figure 1C). Briefly, hADSCs were seeded on six‐well collagen I coated BioFlex™ culture plates. Cyclic stretch was applied in a sinusoidal pattern with 10% amplitude at 0.5 Hz for designated duration (stretch group). Cells cultured in the same plates but left non‐stretched were used as controls (static group). The experimental protocol is summarized (Figure 1D).

### 2.6 Cell survival/viability assay

The surviving cells in each well were manually counted under microscopy. Cell viability was assayed by the Alamar Blue reagent (Thermo Fisher Scientific, Waltham) according to the manufacturer's protocol. The number of viable cells correlated with the magnitude of dye reduction and was expressed as percentage of AB reduction. It was corrected for background values of negative controls containing medium without cells. The percentage of AB reduction is expressed as fold change over the controls.

### 2.7 Flow cytometry for cell cycle, apoptosis and cell size analyses

Cells were sedimented and fixed in 70% ethanol for overnight at 4°C. RNA was digested with RNase A Solution (5 Prime GmbH, Hilden, Germany), and DNA was stained with propidium iodide (Sigma, Deisenhofen, Germany) for 40 min at 37°C. Assessment of cellular apoptosis was done with Annexin V Apoptosis Detection Kit (BD Biosciences, New York). Cell cytrometry analysis was performed using a BD FACSCalibur (BD Biosciences, Heidelberg, Germany) and analysed by FlowJo version 9.3.2 (Tree Star, Inc, Ashland). FSC‐A parameters of the software were used for cell size measurements, as recommended by BD.

### 2.8 Immunofluorescence for Ki67

Cells were fixed in 4% paraformaldehyde in PBS for 20 min at room temperature. Both samples then were washed in PBS, permeabilized, blocked and stained with anti‐Ki67 Primary Antibody (1:1000; Abcam, Cambridge, UK), and then Goat anti‐Rabbit Secondary Antibody, Alexa Fluor 555 (1:500; Invitrogen, Carlsbad, CA, USA). Nuclei were stained using DAPI. Images were visualized using a confocal microscope (Zeiss, Germany) by using 555‐nm excitation and 530‐nm emission. Quantitative analyses were performed by counting the positively stained cells under microscopy within at least five randomly chosen fields.

### 2.9 TUNEL assay

The TUNEL method was performed with the one step TUNEL kit according to the manufacturer's instructions (Beyotime Institute of Biotechnology, China). Briefly, cells were fixed in 4% paraformaldehyde in PBS for 20 minutes at room temperature. Then the cells were permeabilized with 0.3% Triton X‐100 in PBS followed by TUNEL for 1 hour at 37°C. Nuclei were stained using DAPI. Images were visualized under a confocal microscope (Zeiss, Germany) by using 488‐nm excitation and 530‐nm emission. Quantitative analyses were performed by counting the positively stained cells under microscopy within at least five randomly chosen fields.

### 2.10 Assays for cell adhesion and migration

For adhesion assay, cells were plated at a density of 1 × 10^5^ cells/well in a six‐well plate and were incubated for 30 minutes at 37°C. The wells were washed three times, and the number of attached cells was counted under microscope within at least five randomly chosen fields. Adhesion is expressed as fold change over the controls.

Cell migration was assessed using a Transwell Boyden Chamber (pore diameter: 8 μm. Millipore, Billerica, MA) as described previously. The migrated cells on the underside of the filters were counted under a microscope within at least five randomly chosen fields. Migration is expressed as fold change over the controls.

### 2.11 Western blot analysis

Total proteins were extracted from cells utilizing Radio immunoprecipitation assay (RIPA) lysis buffer. After determining the concentrations of protein by bicinchoninic acid (BCA) assay (Thermo Fisher Scientific, Waltham, MA), twenty micrograms total protein fractions were separated by 10% SDS‐PAGE and electroblotted in polyvinylidene difluoride (PVDF) membranes (Millipore, Bedford, MA). The membranes were blocked with 5% BSA and then probed with primary antibodies against integrinβ1, phosphor‐FAK, FAK (1:1000; both from Abcam, Cambridge, UK), cleaved caspase‐3, phosphor‐ERK, phosphor‐AKT, phosphor‐JNK, PI3K (1:1000; all from Cell Signaling Technology, Danvers, MA, USA) and GAPDH (1:10000; Bioworld, MI). These blots were then incubated with HRP‐conjugated secondary antibodies and visualized using the enhanced chemiluminescence detection system (Millipore, Bedford, MA). Quantitative analysis was conducted on immunoreactive bands by Image J software.

### 2.12 Enzyme‐linked immunosorbent assay

After cell stretching for 24 h, the culture medium was collected and centrifuged at 2,500 rpm for 10 min. The supernatants were used and the concentrations of cytokines TGF‐β1, IGF‐1, VEGF, HGF, KGF, bFGF, SDF‐1α and IL‐6 were measured by enzyme‐linked immunosorbent assay (ELISA) kits from the R&D system (Minneapolis, MN, USA) in accordance with the instruction manual. The number of ADSCs were counted and the results have been shown in the unit of “pg/mL/10^5^cells”.

### 2.13 RNA isolation and real‐time polymerase chain reaction

TRIzol reagent (Invitrogen, Mulgrave, Australia) was utilized to extract the total RNA. The RNA was reverse transcribed into complementary DNA by using RevertAid Reverse Transcriptase (Thermo Scientific, Waltham, MA) according to the manufacturer's instructions. Real‐time polymerase chain reaction (RT‐PCR) was performed with SYBR Premix EX Taq (Takara, Dalian, China) by using ViiA 7 (Life Technologies, Carlsbad, CA). Primers used in this study are shown in Table 1. The housekeeping gene GAPDH was used for normalization.

**Table 1:** QRT‐PCR primer sequence

Gene	Primer	Sequences (5′‐3′)
aP2	Forward	ACTGGGCCAGGAATTTGACG
	Reverse	CTCGTGGAAGTGACGCCTT
PPARγ2	Forward	TACTGTCGGTTTCAGAAATGCC
	Reverse	GTCAGCGGACTCTGGATTCAG
adipsin	Forward	TCCAAGCGCCTGTACGAC
	Reverse	GTGTGGCCTTCTCCGACA
LPL	Forward	TCATTCCCGGAGTAGCAGAGT
	Reverse	GGCCACAAGTTTTGGCACC
ALP	Forward	ACTGGTACTCAGACAACGAGAT
	Reverse	ACGTCAATGTCCCTGATGTTATG
COL1a1	Forward	GTGCGATGACGTGATCTGTGA
	Reverse	CGGTGGTTTCTTGGTCGGT
osteopotin	Forward	CTCCATTGACTCGAACGACTC
	Reverse	CAGGTCTGCGAAACTTCTTAGAT
osteocalcin	Forward	CACTCCTCGCCCTATTGGC
	Reverse	CCCTCCTGCTTGGACACAAAG
GAPDH	Forward	ACAACTTTGGTATCGTGGAAGG
	Reverse	GCCATCACGCCACAGTTTC

### 2.14 Quantitative SA‐β‐galactosidase assay

Cells were fixed with 4% paraformaldehyde in PBS, washed with PBS, and then stained using a senescence‐associated (SA) β‐gal staining kit (Cell BioLabs, San Diego, CA) for 24 hours in an incubator chamber at 37°C in the dark. Positive cells were counted and results were expressed as the mean percentage of SA‐β‐gal‐positive cells among total cells.^21^, ^22^

### 2.15 Antibody array

The Phospho Explorer Antibody Array (CSP100) was used to screen for differential expression of more than 130 protein phosphorylation sites (H‐Wayen Biotechnologies, Shanghai, China). Analysis was carried out according to the protocol provided. Briefly, proteins were extracted and labelled by biotinylation. Then biotin‐labelled proteins were applied to the array for conjugation. The conjugation‐labelled protein was detected by Cy3‐Streptavidin. The analysed results were expressed by the ratio of phosphorylation/unphosphorylation.^23^, ^24^

### 2.16 Statistical analyses

Statistical differences among groups were assessed using two‐tailed Student's *t* test. *P* <* *0.05 was considered statistically significant. Results are presented as the mean ± SD.

## 3 RESULT

### 3.1 Flow cytometry and multipotency analysis of isolated hADSCs

hADSCs were isolated from human subcutaneous adipose tissues and expanded easily when cultured in regular medium in vitro, which exhibited morphologically heterogeneous and fibroblastic shape. They were confirmed positive for CD73, CD90 and CD105, negative for CD34, CD45 and HLA‐DR according to flow cytometry analysis of stem cell‐related surface markers (Figure 1A). This is consistent with the typical mesenchymal stem cell surface marker profile. To verify the ability of the generated hADSCs cultures to differentiate into multiple cell types, the cells were analysed for osteogenic, adipogenic and chondrogenic potentials in vitro. This was confirmed by using oil red O staining for lipid droplets formation, alizarin red S staining for calcium deposits formation and Alcian blue staining for sulphated proteoglycans formation (Figure 1B).

### 3.2 Mechanical stretch enhanced the proliferation of hADSCs

**Figure 2:**
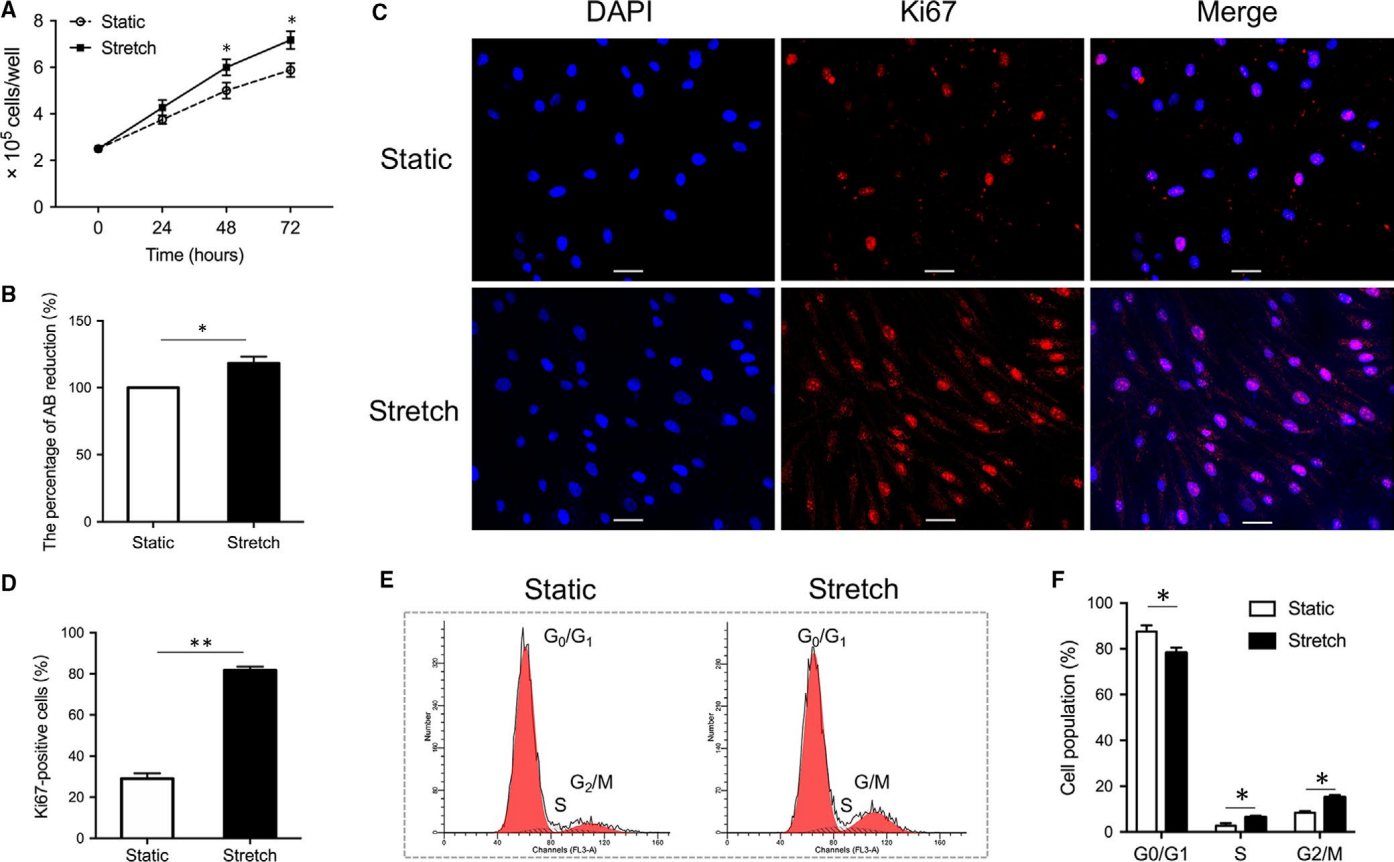
The effect of mechanical stretch on the proliferation of human adipose‐derived stem cells. (A) The number of surviving cells were calculated. *P < 0.05 *vs* static group. (B) Metabolic activity was analysed by Alamar Blue assay. *P < 0.05 vs static group. (C) Proliferative capacity was determined by Ki67 immunofluorescent staining. Scale bar: 20 μm. (D) Quantitative analysis of the percentage of Ki67 positive cells. **P < 0.01 *vs* static group. (E) Cell cycle was analysed by flow cytometry. (F) Quantitative analysis of the cell population in the G_0_/G_1_ phase, S phase and G_2_/M phase of cell cycle. *P < 0.05 vs static group

To investigate the effect of mechanical stretch on the cellular proliferation, hADSCs were cultured under cyclic stretch stimulation for 24 hours to 72 hours. Quantitative analysis showed that the cell numbers were significantly more in the stretch group than controls at all examined time‐points (*P* < 0.05, Figure 2A), although the cell counts were similar between both groups at baseline. Additionally, metabolic activity was analysed by Alamar Blue assay. We found that hADSCs cultured under cyclic stretch stimulation exhibited an increased activity compared with controls (*P* < 0.05, Figure 2B). Furthermore, the cellular proliferative capacity was determined by Ki67 immunofluorescent staining (Figure 2C). Compared to the static group, the stretch group showed a significant increase in Ki67 positive cells (*P* < 0.01, Figure 2D), which indicated that hADSCs cultured under cyclic stretch stimulation clearly exhibited a higher proliferation rate compared with controls. Moreover, flow cytometric analysis of DNA content demonstrated the same trend that mechanical stretch led to a decrease in cell population in the G_0_/G_1_phase and an increase in the S phase and G_2_/M phase (*P* < 0.05, Figure 2E,F). Together, our results indicated that mechanical stretch enhanced the proliferation of hADSCs.

### 3.3 Mechanical stretch inhibited the glucose deprivation induced apoptosis of hADSCs

**Figure 3:**
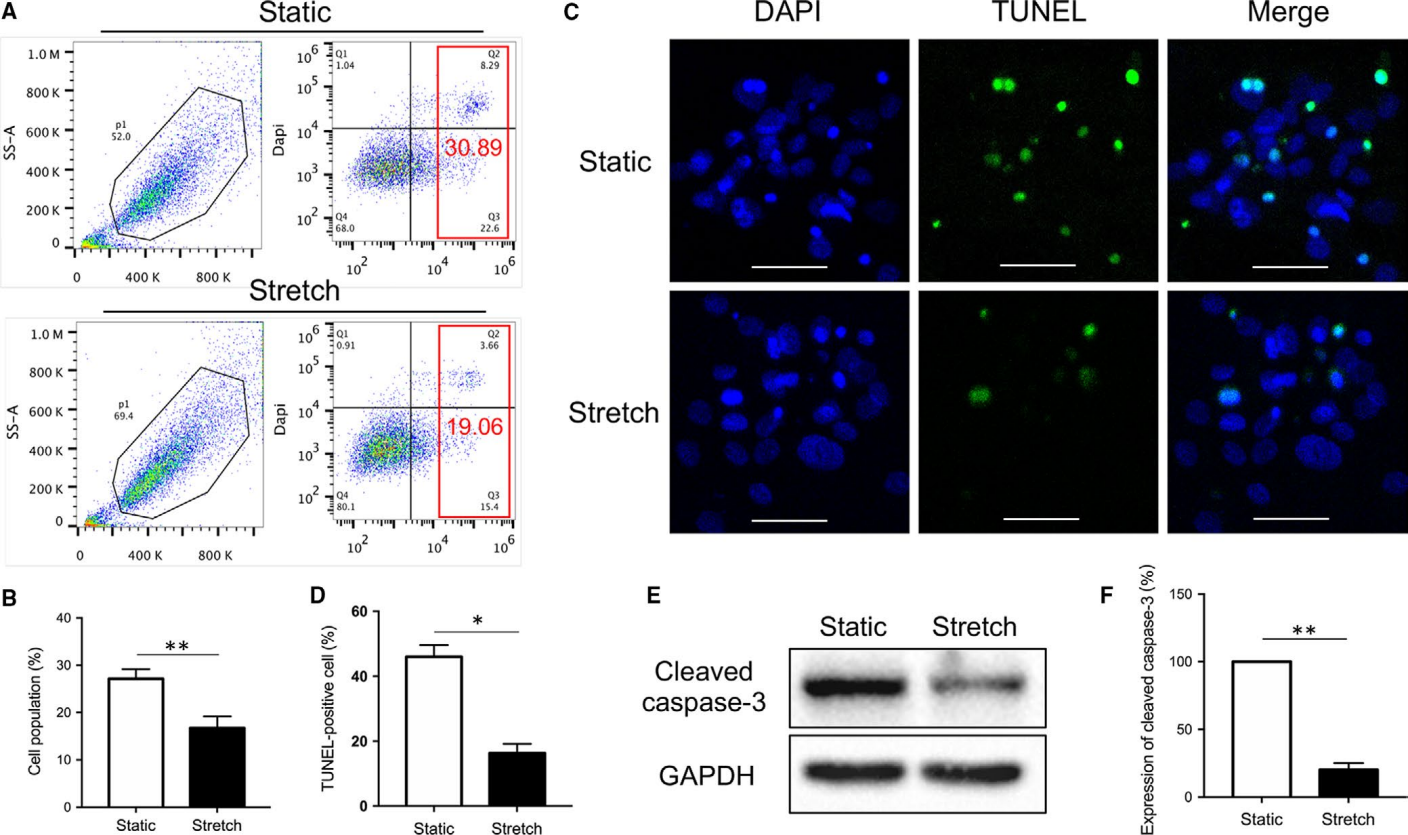
The effect of mechanical stretch on the glucose deprivation induced apoptosis of human adipose‐derived stem cells. (A) The glucose deprivation induced apoptosis was analysed by flow cytometry. (B) Quantitative analysis of the apoptotic cell population. **P < 0.01 vs static group. (C) The glucose deprivation induced apoptosis was evaluated by TUNEL assay. Scale bar: 20 μm. (D) Quantitative analysis of the percentage of TUNEL positive cells. *P < 0.05 vs static group. (E) Expression of cleaved caspase‐3 was determined by Western blotting. (F) Quantitative analysis of the protein bands density of cleaved caspase‐3. The analysis was based on mean grey values normalized to GAPDH. **P < 0.01 vs static group

To explore the effect of mechanical stretch on the glucose deprivation induced cellular apoptosis, hADSCs preconditioned with cyclic stretch for 12 hours, then subjected to glucose deprivation under static conditions for 8 hours. Apoptotic cell population was analysed by flow cytometry (Annexin V staining). Compared to the static group, the stretch group showed a significant decrease in apoptotic cells (*P* < 0.01, Figure 3A,B). In addition, the TUNEL assay was utilized to further validate the apoptotic cell proportion and the result was consistent with the results obtained from flow cytometry assay (*P* < 0.05, Figure 3C,D). Further Western blot analysis showed that the expression of cleaved caspase‐3 was significantly lower in the stretch group than controls (*P* < 0.01, Figure 3E,F). These data suggested that mechanical stretch inhibited the glucose deprivation induced apoptosis of hADSCs.

### 3.4 Mechanical stretch improved the adhesion and migration of hADSCs

**Figure 4:**
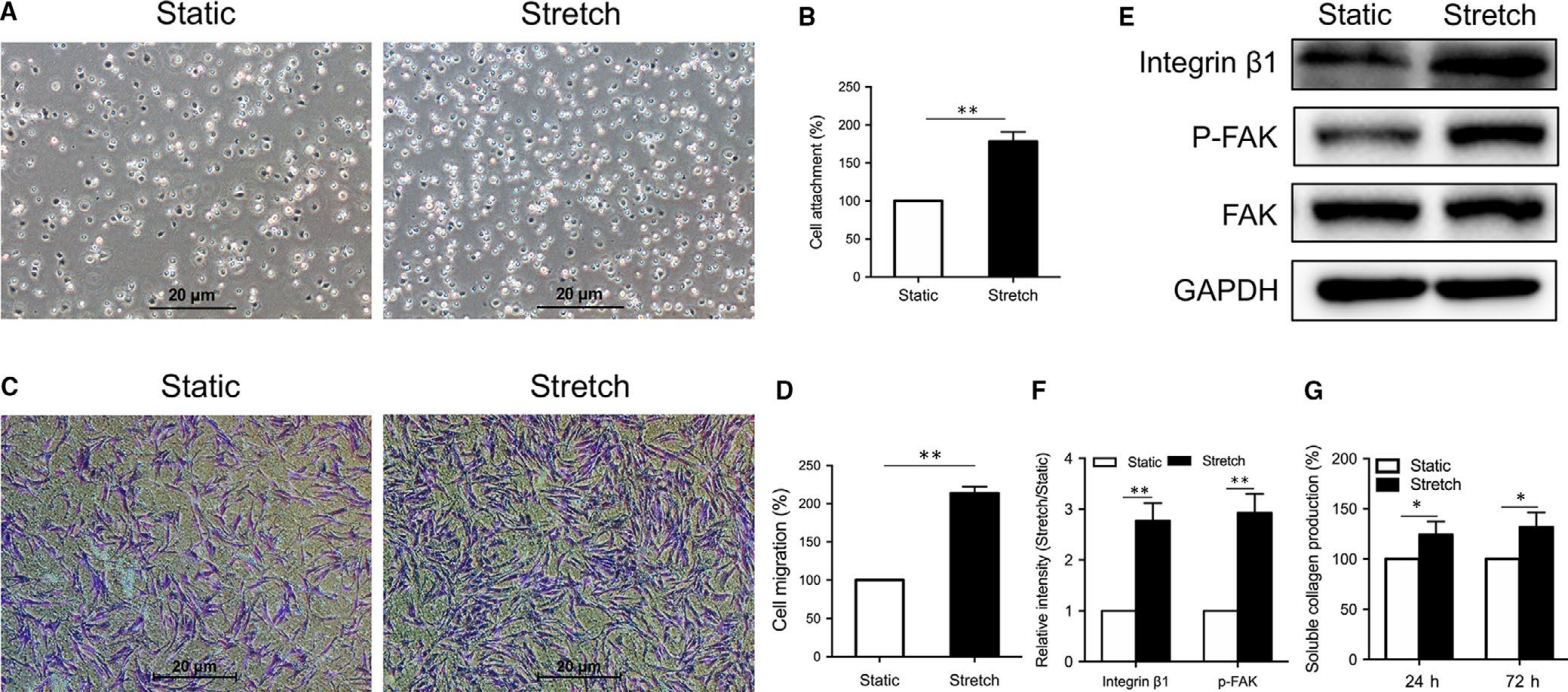
The effect of mechanical stretch on the adhesion and migration of human adipose‐derived stem cells (hADSCs). (A) Representative images of attached hADSCs. Scale bar: 20 μm. (B) Quantitative analysis of the percentage of cell attachment. **P < 0.01 vs static group. (C) Cell migration was conducted using a Transwell Boyden Chamber. Scale bar: 20 μm. (D) Quantitative analysis of the percentage of cell migration. **P < 0.01 vs static group. (E) Expression of the integrin β1, p‐FAK and FAK proteins was determined by Western blotting. (F) Quantitative analysis of the proteins bands density of integrin β1 and p‐FAK. The analysis was based on mean grey values normalized to GAPDH. **P < 0.01 vs static group. (G) The ECM proteins were detected by Collagen Production assay. *P < 0.05 vs static group

We next evaluated the effect of mechanical stretch on the adhesion and migration of hADSCs. hADSCs preconditioned with cyclic stretch for 12 hours were replanted onto coverslips and cultured under static conditions. After 30 minutes of incubation, a portion of the cells were attached to the plate of the well, which adopted a uniform round‐shaped population. More cells were attached in the stretch group compared with controls (*P* < 0.01, Figure 4A,B). The cellular migration was detected using a Transwell Boyden Chamber. After 24 hours of incubation, more cells migrated to the underside of the filters in the stretch group compared with controls (*P* < 0.01, Figure 4C,D). Western blot analysis showed that the expression of integrin‐β1 and p‐FAK, two critical adhesion molecules for cell motility, increased significantly after mechanical stretch (*P* < 0.01, Figure 4E,F). In addition, the expression of the ECM proteins was also enhanced by mechanical stretch (*P* < 0.05, Figure 4G). These findings demonstrated the significant function of mechanical stretch in improving the adhesion and migration of hADSCs.

### 3.5 Mechanical stretch up‐regulated the production of cytokines released from hADSCs

**Figure 5:**
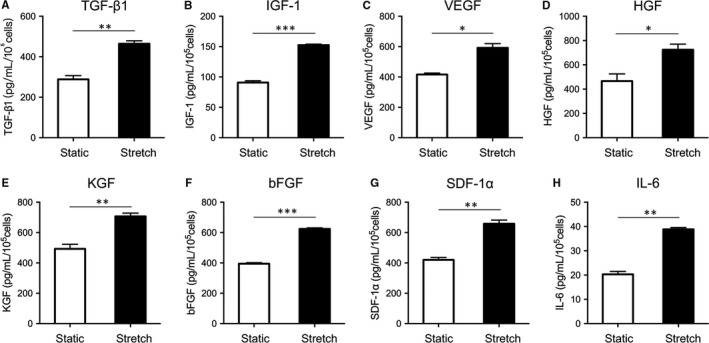
The effect of mechanical stretch on the cytokines released from human adipose‐derived stem cells (hADSCs). The concentrations of pro‐healing cytokines and growth factors released from hADSCs in conditioned media were determined by ELISA: (A) TGF‐β1, (B) IGF‐1, (C) VEGF, (D) HGF, (E) KGF, (F) bFGF, (G) SDF‐1α, (H) IL‐6. *P < 0.05, **P < 0.01 and ***P < 0.001 vs static group

To detect paracrine factors, hADSCs were cultured in serum‐free media under cyclic stretch stimulation. We collected the supernatant after 24 hours for ELISA. The cytokines and growth factors, including TGF‐β1 (*P* < 0.001, Figure 5A), IGF‐1 (*P* < 0.001, Figure 5B), VEGF (*P* < 0.01, Figure 5C), HGF (*P* < 0.05, Figure 5D), KGF (*P* < 0.01, Figure 5E), bFGF (*P* < 0.001, Figure 5F), SDF‐1α (*P* < 0.01, Figure 5G) and IL‐6 (*P* < 0.001, Figure 5H) were up‐regulated in the stretch group compared with controls. These data indicated that mechanical stretch up‐regulated the pro‐healing cytokines and growth factors released from hADSCs, which have an effect in promoting tissue repair and regeneration.

### 3.6 Mechanical stretch inhibited adipogenesis, but enhanced osteogenesis of hADSCs

**Figure 6:**
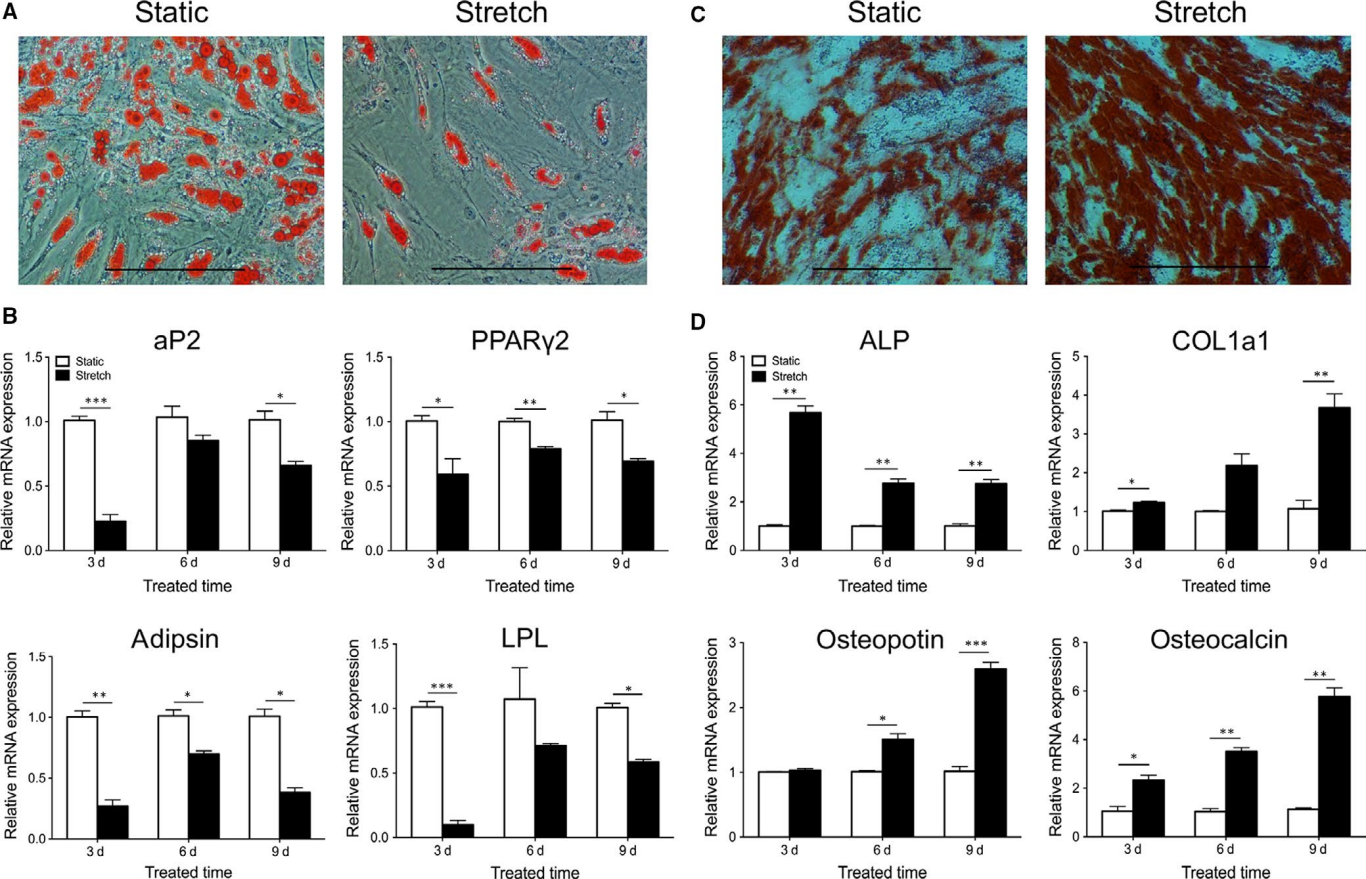
The effect of mechanical stretch on adipogenesis and osteogenesis of human adipose‐derived stem cells. (A) Representative images of lipid droplets with Oil Red O staining after adipogenic induction. Scale bar: 100 μm. (B) Relative mRNA expression (normalized to GAPDH) of aP2, PPARγ2, adipsin and LPL. *P < 0.05, **P < 0.01 and ***P < 0.001 vs static group. (C) Representative images of calcium deposits with Alizarin Red S staining after osteogenic induction. Scale bar: 100 μm. (D) Relative mRNA expression (normalized to GAPDH) of ALP, COL1a1, osteopotin and osteocalcin. *P < 0.05, **P < 0.01 and ***P < 0.001 vs static group

After adipogenic induction, the number of Oil Red O positive lipid droplets was decreased under cyclic stretch stimulation (Figure 6A). In addition, the stretch group possessed lower expression levels of adipocyte fatty acid‐binding protein 2 (aP2), peroxisome proliferator‐activated receptor γ2 (PPARγ2), adipsin and lipoprotein lipase (LPL) (*P* < 0.05, 0.01 or 0.001, Figure 6B). During osteogenic induction, hADSCs cultured under cyclic stretch stimulation expressed higher calcium deposits as shown by Alizarin Red S staining (Figure 6C) and expressed increased level of alkaline phosphatase (ALP), type I collagen (COL1a1), osteopotin and osteocalcin compared with controls (*P* < 0.05, 0.01 or 0.001, Figure 6D). From the above mentioned findings, hADSCs cultured under cyclic stretch stimulation had inhibited adipogenesis, but enhanced osteogenesis.

### 3.7 Long‐term mechanical stretch promoted ageing of hADSCs, but did not alter the cell size and typical immunophenotypic characteristics

**Figure 7:**
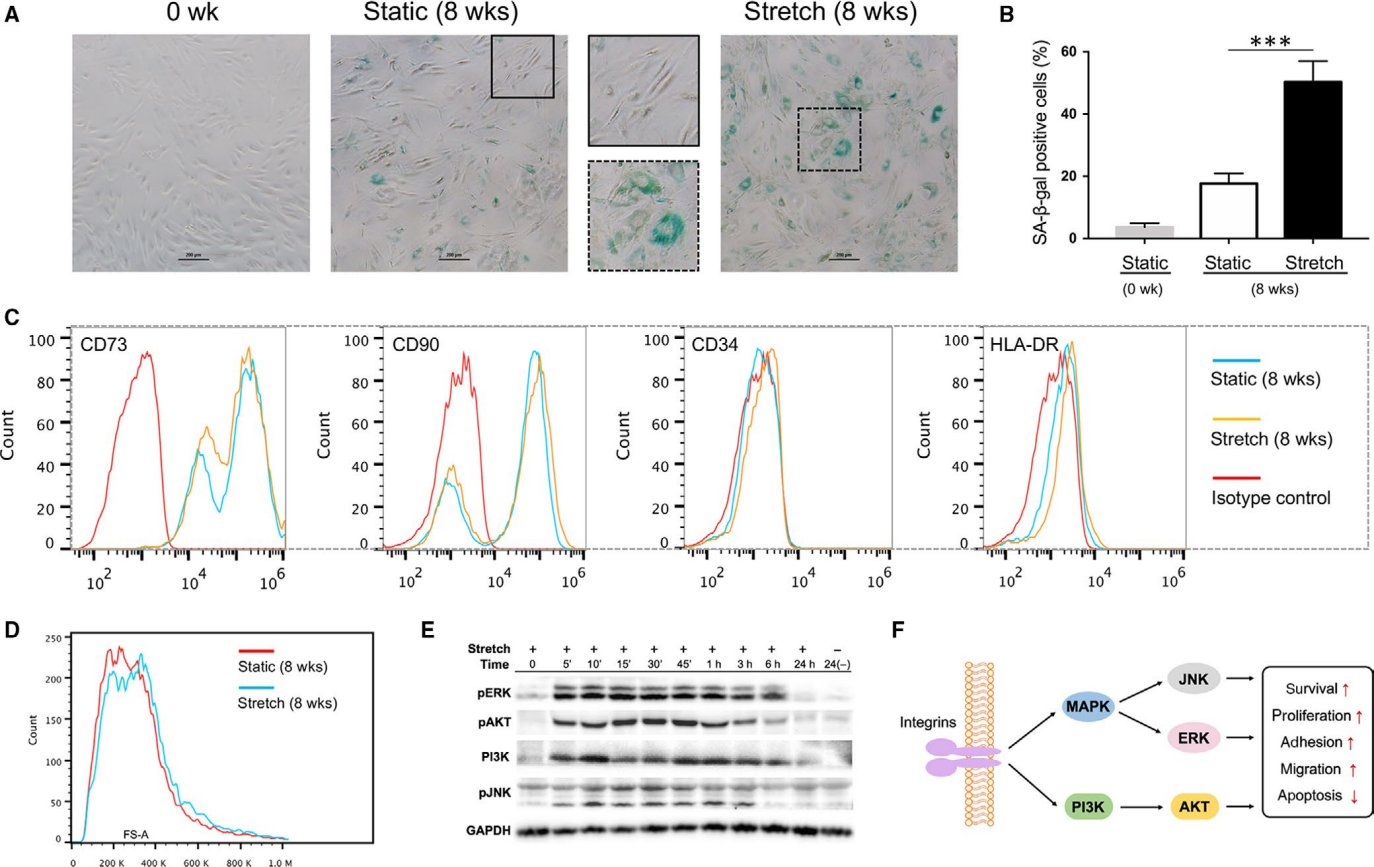
Phenotypic characteristics of human adipose‐derived stem cells (hADSCs) cultured under long‐term cyclic stretch stimulation and investigation of cell signalling events. (A) Senescence‐associated β‐galactosidase (SA‐β‐gal) staining of hADSCs after long‐term cultivation. Scale bar: 200 μm. (B) Quantitative analysis of the percentage of SA‐β‐gal positive cells. ***P < 0.001 *vs* static group. (C) Surface markers were analysed by flow cytometry. (D) Change in cell size was measured using flow cytometry and reflected by the forward‐scatter signal. (E) Expression of p‐ERK, p‐AKT, PI3K and p‐JNK was determined by Western blotting. (F) Proposed schematic diagram of regulation in biological characteristics of hADSCs by mechanical stretch

To study whether long‐term cyclic stretch stimulation affects the biological characteristics of hADSCs, P3 hADSCs were passaged under cyclic stretch stimulation once per week for 8 weeks. We found that more SA‐β‐gal positive cells were detected in hADSCs cultured under mechanical stretch stimulation than controls (*P* < 0.001, Figure 7A,B). In addition, hADSCs were still positive for CD73 and CD90, but negative for CD45 and HLA‐DR as before (Figure 7C). And there were no significant differences in cell size between both groups (Figure 7D). These results implied that long‐term mechanical stretch promoted ageing of hADSCs, but did not alter the cell size and typical immunophenotypic characteristics.

### 3.8 PI3K/AKT and MAPK signalling pathways may participate in the effects of mechanical stretch on the biological characteristics of hADSCs

To research the signalling events, we used a Phospho Explorer antibody microarray covering most typical known signalling pathways. hADSCs were cultured under cyclic stretch stimulation for 30min and the phosphorylation states of proteins were then detected using antibody‐based arrays (Figure S1). We found that activated factors or proteins have been concentrated in PI3K/AKT and MAPK cellular signalling pathways (Tables S1 and S2). To determine whether cyclic stretch could activate the PI3K/AKT and MAPK pathways, which might contribute to the effects of mechanical stretch on the biological characteristics of hADSCs. hADSCs were exposed to cyclic stretching for 0, 5, 10, 15, 30, 45 minutes or 1, 3, 6, 24 hours, and the expression of p‐ERK, p‐AKT, PI3K and p‐JNK was detected by western blot. We observed that the expression of all the proteins above increased rapidly after stretching and peaked at 30 minutes and then gradually fell back to the control level after 6 hours (Figure 7E). These results demonstrated that cyclic stretch stimulation could activate the PI3K/AKT and MAPK signalling pathways in hADSCs.

## 4 DISCUSSION

Nowadays, regenerative medicine as well as tissue engineering is searching for novel methods, which can promote the regenerative process of different tissue injuries and organ damages. Increasing evidence have demonstrated that ADSCs promote tissue repair through both direct regeneration and indirect mechanisms.^25^ In addition, exogenous mechanical stretch has been proved to play an important role in regulating structure and function of various cells.^26^, ^27^ Mechanical stretch pretreatment has been reported to have positive effect on increasing therapeutic efficacy of cell transplantation in tissue engineering, such as dermal fibroblast.^28^ In plastic and reconstructive surgery, synergistically combining ADSCs and mechanical stretch, has been used for skin and adipose regeneration.^29^, ^30^ However, the underlying mechanisms by which synergy between ADSCs and mechanical stretch induced tissue regeneration were not investigated. In this study, we explored the effects of mechanical stretch on the biological characteristics of hADSCs, including proliferation, apoptosis, adhesion and migration, differentiation, cytokine production and phenotypic characteristics during long‐term cultivation.

First of all, we analysed the survival and viability that are most important factors directly correspond with regenerative process.^31^ We found that hADSCs cultured under cyclic stretch stimulation exhibited significantly elevated proliferative activity and metabolic viability. Obtained data stands in good agreement with previous findings in human bone marrow mesenchymal stem cells and bovine aortic endothelial cells.^32^, ^33^ Therefore, the application of mechanical stretch could be an important strategy for promoting proliferation of ADSCs in cell therapies that require good survival rates of the transplanted cells. Owing to the ischaemia and anoxia in recipient site, apoptosis remains the biggest hurdles for transplantation of MSCs.^34^, ^35^ When hADSCs were preconditioned with cyclic stretch, the glucose deprivation induced apoptosis was significantly compromised in our study. But previous studies have shown that excessive stretch itself also induces apoptosis,^36^ which can be explained by the excessive extension and frequency. In other words, cyclic stretch at certain extent of parameters is important for the observed protective effects.

Investigators have explored ways to increase MSCs migration and adhesion into injuries portion to promote tissue repair.^37^ In the current study, we found that mechanical stretch improved the adhesion and migration of hADSCs. FAK and integrin‐β1 are two adhesion molecules which are known to be critical for cell motility.^38^, ^39^ In our study, mechanical stretch could activate the FAK signals and up‐regulate the expression of integrin‐β1 in hADSCs, which are consistent with previous studies in cardiomyocytes and vascular smooth muscle cells.^40^, ^41^ What is more, the expression of the ECM proteins was enhanced in the stretch group, which is also important to cell survival and motility.^42^ Our study confirmed the function of mechanical stretch on the adhesion and migration of hADSCs, which may be a novel method to increase MSCs delivery and efficacy.

A growing body of evidence have illustrated that the effect of promoting wound healing by ADSCs partly attributed to the production of diverse growth factors.^43^ Previous studies showed that conditioned medium of ADSCs (ADSCs‐CM) has the ability to promote tissue regeneration.^44^ Our data showed that many kinds of tissue regeneration related cytokines, including TGF‐β1, IGF‐1, VEGF, HGF, KGF, bFGF, SDF‐1α and IL‐6 were up‐regulated in the stretch group. Therefore, mechanical stretch may be an alternative preconditioning method of ADSCs‐CM. Recent studies have indicated that a plethora of mechanical stimuli contribute to the normal development of organisms, including mechanical stretch concomitant with physical movement.^45^, ^46^ In the current study, we found that mechanical stretch maintained the differentiation potentials of hADSCs, but differently regulated the adipogenic and osteogenic differentiation of hADSCs. Mechanical stretch inhibited adipogenesis, but promoted osteogenesis, which is in line with previous research in rat bone marrow MSCs.^47^ These results may provide a scientific basis for the application of improved exercise therapy regimens in the treatment of metabolic obesity and osteoporosis.

Phenotypic characteristics of hADSCs under cyclic stretch stimulation during long‐term cultivation was also investigated. We observed a senescent phenotype characterized by enlarged and flattened cell morphology, and the expression of SA‐β‐gal during long‐term culture of hADSCs under cyclic stretch stimulation. By contrast, ADSCs cultured under static condition maintained the phenotypic characteristics of early passage hADSCs to a great degree. However, there were no significant differences in cell size between both groups. It was not surprising that hADSCs in the stretch group aged faster than that in controls, because transient survivable disruptions of cell plasma membrane integrity are now known to occur during tediously long‐time stretch, which may cause cell injury and ageing.^48^ Additionally, mechanical stretch maintains the basic characterization of hADSCs, showing specific surface marker expression. In other words, long‐term stretch stimulation did not carry off the ‘stemness' character of hADSCs.

It has been reported that the transduction pattern of external mechanical signals into the intracellular biological signals determines the cell fate.^49^, ^50^ However, the complete pathways relating specific mechanical stimuli to stem cell fate remain to be elucidated. In the present study, we found that cyclic stretch stimulation could activate the PI3K/AKT and MAPK signalling pathways in hADSCs. PI3K and MAPKs have been reported to play vital roles in regulating mechanotransduction mechanisms.^51^, ^52^ The PI3K/AKT signalling has been identified to regulate stem cell growth, proliferation, differentiation, motility and intracellular trafficking.^53^ MAPKs are specific serine/threonine protein kinases that are involved in regulation of cell growth and differentiation, inflammation and apoptosis.^54^ It is noteworthy that the crosstalk between PI3K/AKT and MAPK signalling pathways is important to regulate stem cell fate.^55^

Taken together, we clearly demonstrated that mechanical stretch significantly promoted the proliferation, adhesion and migration of hADSCs, suppressing cell apoptosis and increasing the production of pro‐healing cytokines. For differentiation of hADSCs, mechanical stretch inhibited adipogenesis, but enhanced osteogenesis. Long‐term stretch could promote ageing of hADSCs, but did not alter the cell size and typical immunophenotypic characteristics. Furthermore, we revealed that PI3K/AKT and MAPK pathways might participate in the effects of mechanical stretch on the biological characteristics of hADSCs. This is the first time (to our knowledge) that the in vitro cyclic stretch model was successfully used to investigate how mechanical stretch affects the multitudinous biological characteristics of hADSCs. These findings provide the first direct evidence that mechanical stretch is an effective strategy for regulating stem cell fate and could serve as an important newfangled pretreatment in stem cell therapy and regenerative medicine.

## CONFLICT OF INTEREST

The authors confirm that there are no conflicts of interest.
